# MADVAR: a lightweight, data-driven tool for automated feature selection in omics data

**DOI:** 10.1093/bioadv/vbaf211

**Published:** 2025-09-04

**Authors:** Gilad Silberberg

**Affiliations:** Department of Bioinformatics, Champions Oncology Inc., Rockville, Maryland, MD 20850, United States

## Abstract

**Motivation:**

High-throughput biological data provides rich opportunities for discovery, but its vastness leads to the inclusion of many irrelevant features that hinder effective analysis, especially in unsupervised clustering and machine learning tasks. Traditional feature selection methods such as correlation filtering, PCA, mutual information, and Laplacian scores often either eliminate important features or demand extensive computational resources, and their thresholds are usually arbitrary rather than data-driven.

**Results:**

MADVAR addresses these challenges as a lightweight R package for automated feature selection in omics data, introducing two data-driven methods—madvar and intersectDistributions—that define thresholds based on the statistical structure of the data itself. These approaches eliminate the reliance on arbitrary cutoffs and efficiently filter features without expensive computation. Benchmarking across diverse omics datasets shows that MADVAR achieves top performance in clustering and classification tasks while maintaining computational efficiency, and it integrates seamlessly into existing R-based analysis pipelines.

**Availability and implementation:**

The source code and documentation for MADVAR are freely available on GitHub (https://github.com/Champions-Oncology/MADVAR). The package is implemented in R and runs on all major operating systems.

## 1 Introduction

High-throughput biological data presents both an opportunity and a challenge: while its diversity and abundance enhance research, it also generates a vast number of irrelevant features, complicating storage, computation, and downstream analysis. This challenge becomes particularly evident in unsupervised clustering and plotting, and feature selection in machine learning (ML) tasks. It also poses a difficulty in the study of biological systems, as only a minority of the detected genes are involved in the perturbed process.

Common approaches for feature selection include filtering highly correlated features, utilization of PCA to retain features with high eigenvalues in the components explaining the highest amount of variance (e.g. Horn’s Parallel Analysis, Horn 1965), mutual information of Laplacian scores calculated for the features, as well as variance-based methods. However, most of these methods suffer from major inherent limitations: (i) correlation-based filtering would likely eliminate many informative features in the system, because they correlate with others; (ii) all approaches mentioned but the variance-based, perform matrix construction for the features and have O(m^2) or O(m^3) complexities. For a large dataset like The Cancer Genome Atlas (TCGA) gene expression, with >48K features and >1.3K samples, a feature matrix is estimated to take up >17 GB of memory and run time could take hours to days; and (iii) cutoffs applied on the calculated metrics are often arbitrary quantitative or percentile-based, and not data-driven.

In this study, two quick and lean variance-based methods, feature variance ranking and variance-mean modelling (“modelGeneVar,” Lun and Marioni 2016), were integrated with novel workflows for automatic data-driven thresholding and benchmarked with other workflows. Two of the best performing workflows were implemented in the MADVAR package, madvar and intersectDistributions. madvar constitutes a complete workflow for feature selection. Its key innovation lies in its ability to automatically determine a variance cutoff based on the statistical properties of the data distribution—specifically the median and median absolute deviation (MAD). I have observed that the variances of most quantitative biological data types are characterized by a right-skewed distribution (or a Poisson distribution with a low λ), stemming from the sparse expression of a substantial proportion of features in a given system. Leveraging this property, I developed an algorithm that automatically detects and excludes the near-zero variance peak, which corresponds to invariant data features, by using the sum of the median and MAD of the distribution. This approach is inspired by the MAD method for outlier detection, where two MADs around the median are used to flag outliers—a concept analogous to the widely used two standard deviations around the mean criterion (described in https://eurekastatistics.com/using-the-median-absolute-deviation-to-find-outliers/). Another novel approach for identifying the near-zero peak was to apply gaussian mixture modelling (GMM) on the variance (or other predictive score), whereby one of fitted distributions models the near-zero peak. intersectDistributions then finds the intersection point on the x axis between the two detected distributions, which in turn can serve as a cutoff.

## 2 System and methods

Run time estimates were calculated for a @ 2.50 GHz core. Datasets were filtered to retain complete rows (features) for the samples used in the study, and the samples selected represent the most frequent metadata groups (Table 1, available as supplementary data at *Bioinformatics Advances* online). RNAseq reads data were variance stabilizing transformation (VST)-normalized (Love *et al.* 2014). Relative abundance matrices were scaled to ≥0 for modelGeneVar, as it assumes log-normalized expression values.

**Table 1.** Percentage of features selected in different cutoff methods.^a^

Dataset	Total features	MADVAR	Variance Elbow	Variance Knee	Variance GMM	MVmodel	MVmodel Madvar	MVmodel Elbow	MVmodel Knee	MVmodel GMM
**TCGA**	48 128	22.86	0.004	0.16	27.16	10.07	10.31	0.015	0.98	20.25
**GTEX**	4633	20.76	0.345	3.35	26.96	19.53	11.5	0.173	3.35	27.65
**CPTAC**	1181	13.46	0.931	1.69	37.17	10.42	11.77	0.169	13.12	23.37

a The “MVmodel” score was filtered by FDR ≤ 0.05. Data types used were: RNAseq VST-normalized counts, Relative protein abundance and Phosphoproteomics from TCGA, GTEX, and CPTAC, respectively.

MAD is calculated as:


$$\mathrm{MAD}=\mathrm{median}(\left|x_{i}-\mathrm{median}(x)\right|),$$

where *x_i_* are the individual data points, and MADVAR cutoff is calculated as:


$$\mathrm{MADVA}R_{\mathrm{cutoff}}=\mathrm{median}(x)+\mathrm{mads}*\mathrm{MAD},$$

where the coefficient mads = 2 by default but can be adjusted by the user.The intersection point between two normal distributions was calculated by equating their weighted density functions:


$$\lambda_{1}\cdot \varphi \left(x{;\mu }_{1}{,\sigma }_{1}\right){=\lambda }_{2}\cdot \varphi \left(x{;\mu }_{2}{,\sigma }_{2}\right),$$

where φ(*x*; μ, σ) is the probability density function of a normal distribution: φ(*x*; μ, σ) = (1/√(2πσ2)) · exp(−(*x*  −  μ)^2^/(2σ^2^)).Taking the logarithm of both sides and rearranging terms yields a quadratic equation of the form: *A*·*x*^2^ + *B*·*x* + C = 0, where the coefficients A, B, and C are derived from the means (μ_1_, μ_2_), variances (σ_1_^2^, σ_2_^2^), and mixing proportions (λ_1_, λ_2_) of the two components in the mixture model.

Euclidean distance and the Ward.D clustering method (Murtagh and Legendre 2014) were used for hierarchical clustering. Random forest was run with ntree = 100. Analyses and plots were done using the R computing environment (R Core Team 2023).

## 3 Implementation

The madvar and the intersectDistributions functions are implemented in the MADVAR R package, available at https://github.com/Champions-Oncology/MADVAR. madvar’s primary argument, data, accepts either a matrix with features as rows or a precomputed variance vector. The function automatically detects the input type and returns either a filtered matrix or the corresponding variance cutoff value, respectively. In addition to the data input, madvar accepts the argument “mads,” which is the coefficient of the MAD (defaults to 2), and returns the filtered dataset or a variance cutoff. It also provides an exploration mode, where the variance density overlaid by the calculated cutoff is plotted. This feature allows the user to visualize the resulting cutoff with respect to the near-zero peak and adjust the “mads” argument if desired. Additionally, madvar offers the option to provide a “must genes” list via the “must_genes” argument, enabling the user to specify features (column names) that should not be filtered out and retained in the output.

Intersect Distributions has a single argument, a mixEM object created by the normalmixEM function in the “mixtools” R package (Benaglia *et al.* 2009), and returns the *x*-value of the intersection between the two underlying distributions modelled by normal mixEM.

## 4 Results

**Figure 1.**
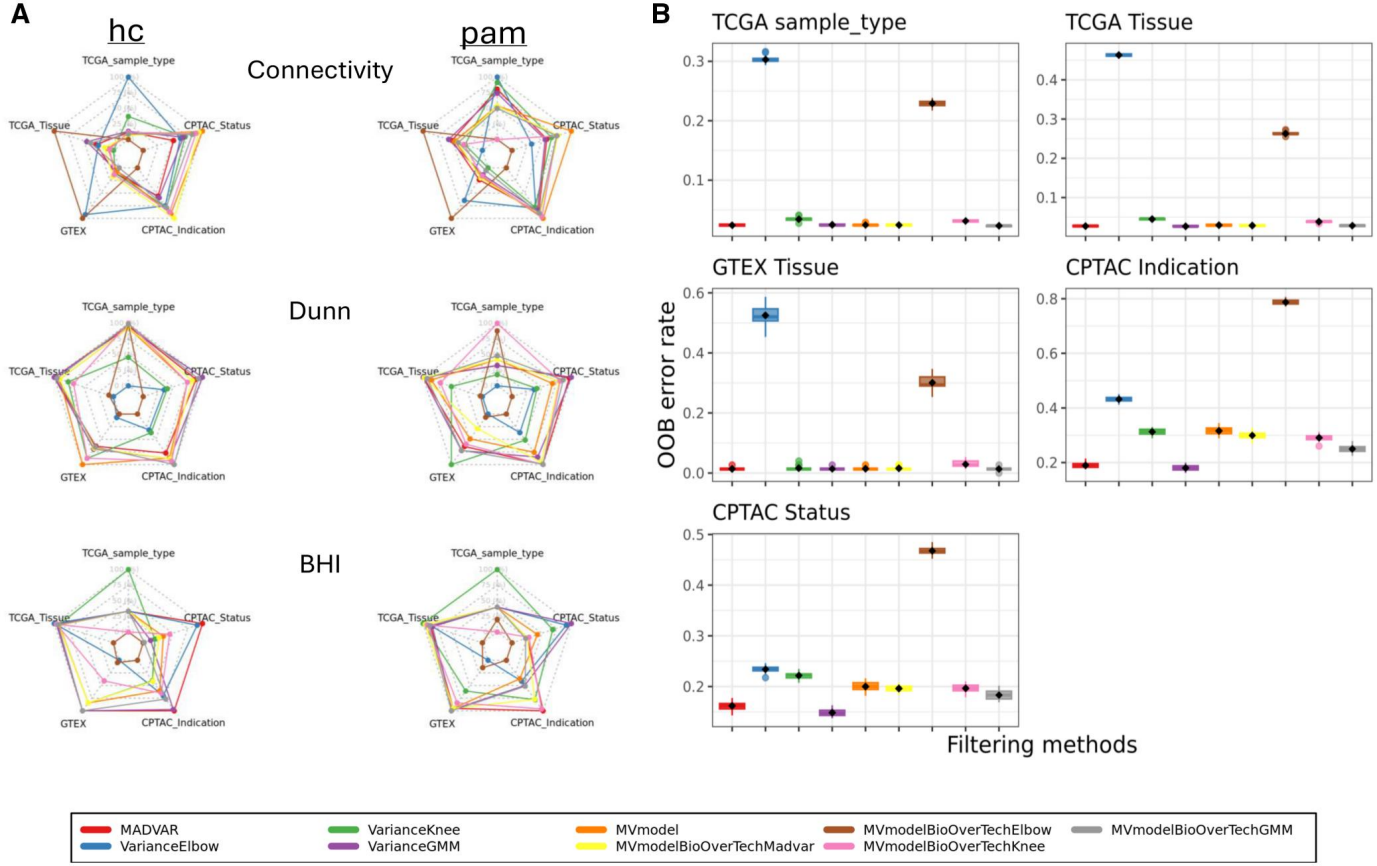
Performance evaluation of MADVAR compared to other variance-based methods and filters. (A) Radar charts for unsupervised clustering. The values are min-max scaled per plot. Note that for the connectivity metric, lower values indicate better connectedness. hc: hierarchical clustering; pam: partitioning around medoids. (B) Out-of-bag (OOB) error rates measured in multiple runs of random forest (*n*=32). The diamond inside the box indicates the mean.

To demonstrate the property of right-skewed variance distribution in various molecular abundance data, multiple datasets of diverse types were collected, including RNAseq normalized counts from TCGA, tandem mass tag (TMT)-labeled proteomics abundance from the Genotype-Tissue Expression (GTEx) portal (GTEx Consortium 2015) and phosphoproteomics data from the clinical proteomic tumor analysis consortium (CPTAC, Zhang *et al.* 2016). When visualizing the feature variance density, all data types displayed similar right-skewed distributions, where the peak on the left end corresponds to near-zero variance (Fig. 1, available as supplementary data at *Bioinformatics Advances* online). Consistently, the mode of the variance corresponded to the summit of the peak, while the median was immediately after, and the madvar cutoff (red) automatically marking the end of the non-zero peak (Fig. 1A, available as supplementary data at *Bioinformatics Advances* online). This demonstrates that madvar can consistently and accurately identify the border of the near-zero peak and exclude the invariant features. Mixed-model fitting of the feature variance distribution is shown in Fig. 1B, available as supplementary data at *Bioinformatics Advances* online, where two modelled underlying density distributions are plotted, along with the x-axis intersection between them (vertical blue line).

To evaluate the relevance of features selected by these approaches, additional combinations of scoring methods and automated thresholding techniques were applied on the three datasets, all of which are computationally quick (2.4 s for madvar and up to 3 s for the other approaches on the TCGA cohort). Besides feature variance, another scoring method was used: mean-variance trend modelling, which decomposes the genes variance into biological (bio) and technical (tech) components (referred to here as “MVmodel”). Its relevance to the current task stems from the rationale that it can identify genes whose variance truly stems from the biology and are relevant to the system. The function “modelGeneVar” in the “scran” package (Lun and Marioni 2016), which implements this algorithm, also calculates raw and adjusted *P*-values for the test against the null hypothesis that bio≤0. The thresholding used for the core MVmodel approach here was FDR ≤0.05, which is the default in many of the package functions. Two additional automatic thresholding methods, “elbow” (maximum second derivative of the ranked score) and “knee” (implemented in the “kneedle” package, Satopaa *et al.* 2011) were applied to the variance and to the ratio bio/tech obtained from “modelGeneVar,” which also showed a right-skewed distribution. In addition, the madvar cutoff and GMM were also calculated on this ratio, thus creating a total of nine feature filters (Table 1). It is apparent that the GMM methods typically retain the highest number of features, with madvar second and the elbow method often retaining very few features, which could not explain by themselves the perturbed system.

The different feature sets were then benchmarked by five clustering tasks (Table 1, available as supplementary data at *Bioinformatics Advances* online) and comparing several performance metrics among them. To assess and compare the quality of unsupervised clustering, we calculated connectivity, Dunn index and Biological Homogeneity Index (BHI) in the different datasets (Fig. 1A). The connectivity index indicates the degree of connectedness of the clusters, as determined by the k-nearest neighbors, has a value between 0 and infinity and should be minimized. The Dunn Index is the ratio of the smallest distance between observations not in the same cluster to the largest intra-cluster distance, has a value between zero and infinity, and should be maximized. BHI measures the average proportion of sample pairs that are clustered together which have matching annotation classes. The BHI is in the range [0,1], with larger values corresponding to more homogeneous clusters. Unsupervised methods used were hierarchical clustering and Partitioning Around Medoids (PAM) clustering, where the number of clusters (k) was applied according to the number of levels in each grouping term (Table 1, available as supplementary data at *Bioinformatics Advances* online). Clustering of MADVAR-filtered, as well as of variance-GMM-filtered data overall were the best performing and most stable, with MADVAR peaking in the BHI, yielding the most homogeneous clusters, and variance-GMM in the Dunn index, generating the most distinguishable clusters (Fig. 1A and Fig. 2, available as supplementary data at *Bioinformatics Advances* online).

To compare classification performance of supervised learning, random forest was chosen as a representative machine learning (ML) method. It was run in multiple seed iterations (*n* = 32), from which the out-of-bag (OOB) error rates were extracted. OOB error estimates the performance of the random forest model using samples not included in the bootstrap sample for training and was used as a performance metric. Here too, MADVAR and variance-GMM stood out in their performance and stability, displaying the lowest mean or median error rate in most all classification tasks (Fig. 1B).

## 5 Discussion

MADVAR enables a faster analysis with a reduced memory requirement, while improving clustering and prediction results with minimal loss of relevant features and implements computationally fast and lean applications, practical for large datasets. Conceptually, it optimizes the balance between feature selection, an essential step in ML approaches, and the integrity of data elements required to explain a complete biological system. Tasks such as mechanism of action (MoA) interrogation or regulatory network reconstruction can therefore greatly benefit from MADVAR feature filtering as it effectively reduces the dimensionality of high-throughput biological data by excluding invariant features. This not only enhances the accuracy and efficiency of downstream analyses but also ensures that the most relevant and variable features are retained, thereby improving the interpretability and reliability of the results. Importantly, MADVAR applications are able to automatically apply a data-driven cutoff, eliminating the need for arbitrary thresholds and enhancing reproducibility across datasets.

MADVAR is particularly valuable for researchers working with high-dimensional quantitative biological data, such as transcriptomics and proteomics datasets, but can be extended to other right-skewed data types, such as the bio/tech ratio as demonstrated here. Its implementation in R makes it accessible to the bioinformatics community, and its integration into existing analysis pipelines is straightforward.

In addition to the madvar method, the MADVAR package includes intersectDistributions, which applies GMM to identify the near-zero variance peak and determine a cutoff based on the intersection of two fitted distributions. This method provides an alternative, model-based approach to thresholding and was also benchmarked in the study. While intersectDistributions performed well in clustering and classification tasks, a minor caveat is that it assumes exactly two normal distributions, which may not always capture the complexity of the data. Nevertheless, its performance was robust, particularly in larger datasets where the assumption holds more reliably.

The benchmarking results further support the utility of MADVAR. Across five clustering tasks, MADVAR-filtered- and variance-GMM-filtered data, achieved the best overall ranking across multiple metrics (Fig. 2, available as supplementary data at *Bioinformatics Advances* online). In supervised classification tasks using random forest, both MADVAR and variance-GMM demonstrated comparably low out-of-bag (OOB) error rates, indicating strong and stable predictive performance. Notably, Table 1 suggests that methods retaining a larger number of features tended to perform better in the benchmark, although MADVAR often showed comparable performance to that of variance-GGM, while utilizing considerably fewer features.

## Supplementary Material

vbaf211_Supplementary_Data

## Data Availability

The datasets were derived from sources in the public domain: TCGA RNAseq data (https://portal.gdc.cancer.gov/), GTEx proteomics data (https://gtexportal.org/home/), CPTAC phosphoproteomics data (https://proteomics.cancer.gov/data-portal).
