# Supplementary material for: MADVAR: a lightweight, data-driven tool for automated feature selection in omics data
Source: Bioinform Adv. 2025 Sep 4;5(1):vbaf211. doi: 10.1093/bioadv/vbaf211 (PMC12449246; doi:10.1093/bioadv/vbaf211)
Supplement: vbaf211_Supplementary_Data [file vbaf211_supplementary_data.zip › Table S1.docx]

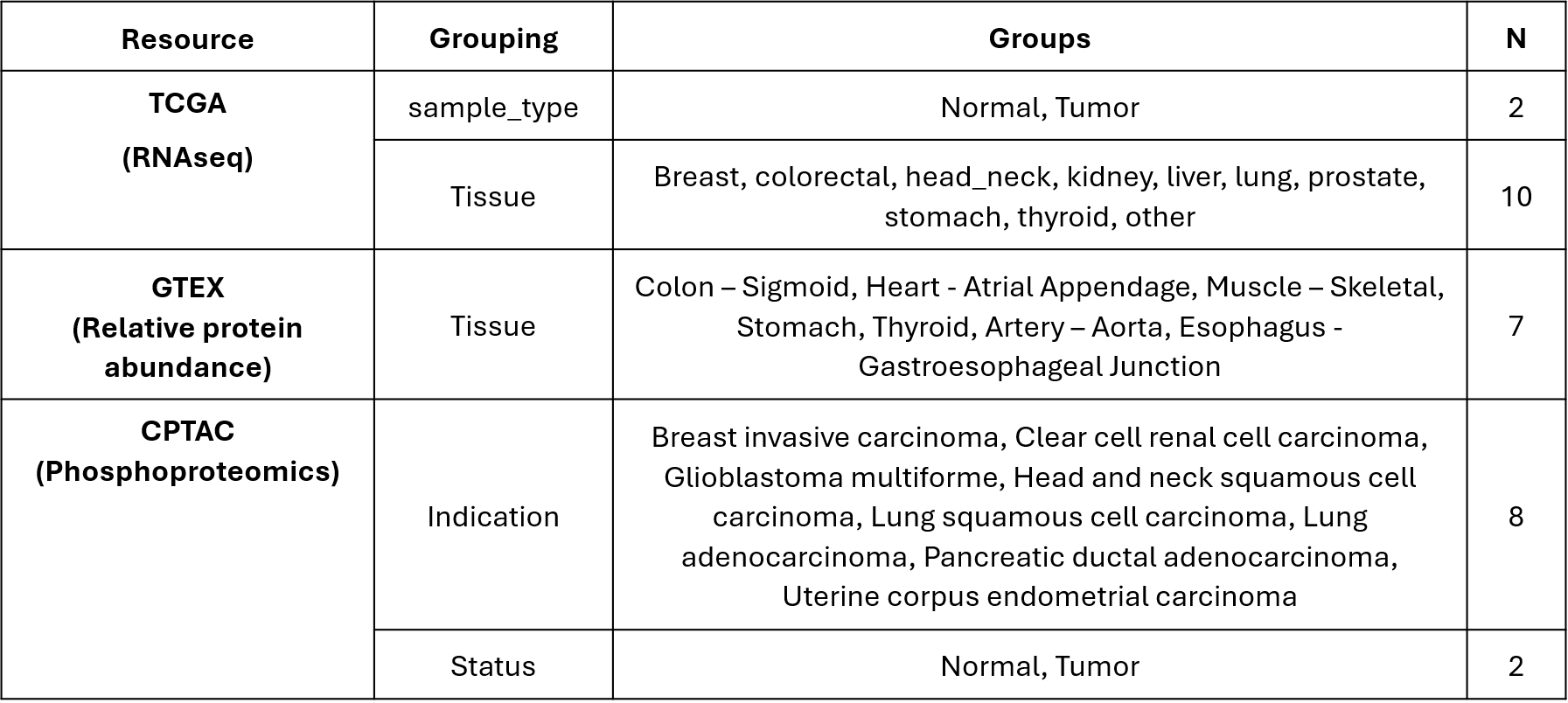


Table S1. Summary of phenotypic grouping in the TCGA cohort used in the study. The number of levels (N) was used as k (number of clusters) in unsupervised clustering assessment.
