# Supplementary figures and images for: MADVAR: a lightweight, data-driven tool for automated feature selection in omics data
Source: Bioinform Adv. 2025 Sep 4;5(1):vbaf211. doi: 10.1093/bioadv/vbaf211 (PMC12449246; doi:10.1093/bioadv/vbaf211)

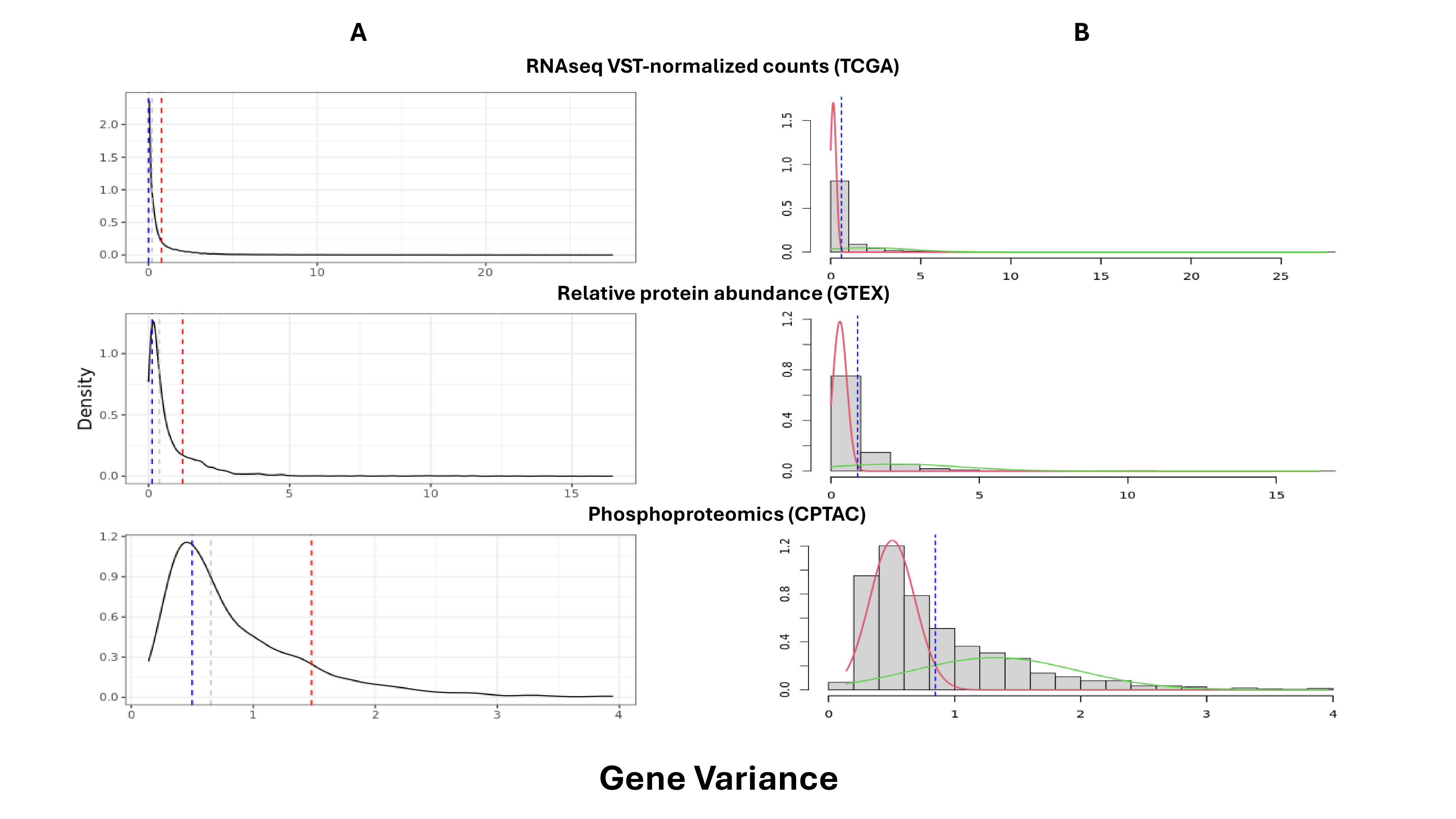

Supplement: vbaf211_Supplementary_Data [file vbaf211_supplementary_data.zip › Supp_Figure1.tif]

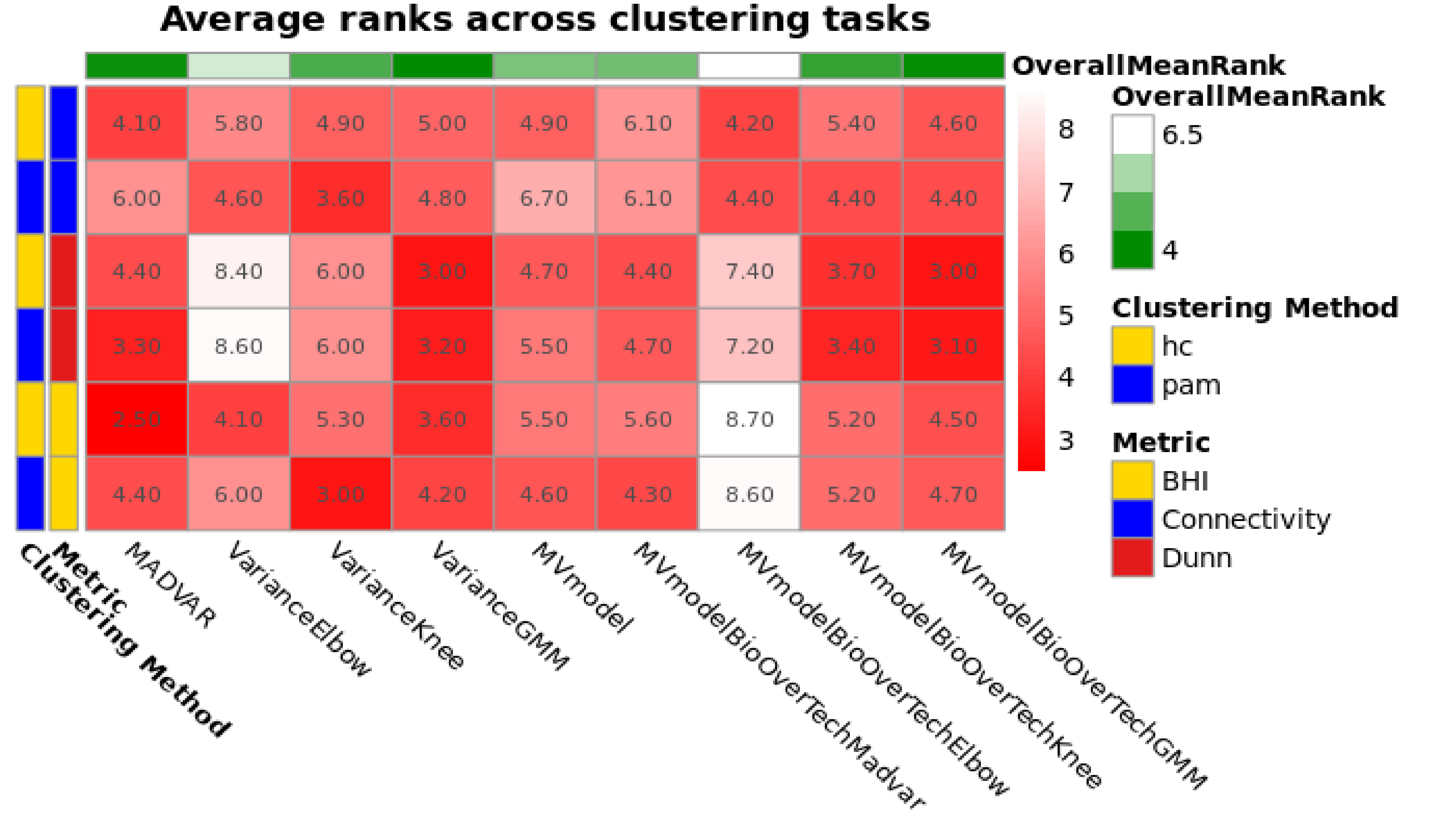

Supplement: vbaf211_Supplementary_Data [file vbaf211_supplementary_data.zip › Supp_Figure2.tif]
